# The antiangiogenic effects of polyisoprenylated cysteinyl amide inhibitors in HUVEC, chick embryo and zebrafish is dependent on the polyisoprenyl moiety

**DOI:** 10.18632/oncotarget.11908

**Published:** 2016-09-08

**Authors:** Augustine T. Nkembo, Elizabeth Ntantie, Olufisayo O. Salako, Felix Amissah, Rosemary A. Poku, Lekan M. Latinwo, Nazarius S. Lamango

**Affiliations:** ^1^ Division of Basic Pharmaceutical Sciences, College of Pharmacy and Pharmaceutical Sciences, Florida A&M University Tallahassee, Tallahassee, Florida 32307, USA; ^2^ Department of Biological Sciences, College of Science and Technology, Florida A&M University Tallahassee, Tallahassee, Florida 32307, USA

**Keywords:** angiogenesis, PCAIs, HUVEC, cell invasion, zebrafish

## Abstract

Angiogenesis is essential for solid tumor growth, therapeutic resistance and metastasis, the latest accounting for 90% of cancer deaths. Although angiogenesis is essential for the malignant transformations in solid tumors and therefore is an attractive target, few drugs are available that block tumor angiogenesis. The focus has been to block signaling by receptor tyrosine kinases (RTKs), such as for vascular endothelial growth factor (VEGF), whose activation abrogate apoptosis and promote angiogenesis. The polyisoprenylated cysteinyl amide inhibitors (PCAIs) were designed to modulate aberrant polyisoprenylated small G-proteins such as mutant Ras whose constitutive activation promotes RTKs signaling. Since polyisoprenylation is essential for protein-protein interactions and functions of G-proteins, we hypothesized that the PCAIs would disrupt the monomeric G-protein signaling thereby effectively inhibiting angiogenesis. In this study we determined the effects of PCAIs on human umbilical vein endothelial cells (HUVEC) tube formation, cell viability, cell migration and invasion as well as *in vivo* using the chick chorioallantoic membrane (CAM) and zebrafish models. At sub- to low micromolar concentrations, the PCAIs inhibit the native and VEGF-stimulated cell migration and invasion as well as tube formation and angiogenesis in CAM and zebrafish embryos. The concentrations that block the angiogenic processes were lower than those that induce cell death. Since angiogenesis is essential for tumor growth but otherwise limited to wound healing, feeding fat cells and uterine wall repair in adults, it is conceivable that these compounds can be developed into safer therapeutics for cancers and retinal neovascularization that leads to loss of vision.

## INTRODUCTION

Angiogenesis, the process by which new blood vessels form, is essential for tumors continued growth as well as metastasis [1]. This is necessary for the supply of sufficient amounts of nutrients and oxygen to sustain cancer cell proliferation and the removal of waste [2, 3]. While normal angiogenesis is widespread in developing organs, it occurs only in a limited number of physiological processes such as in wound healing and uterine thickening during the menstrual cycle in adults [4, 5]. Adult vasculature is stable under normal physiological conditions whereby a balance between pro- and anti-angiogenic factors orchestrates the equilibrium [6]. However, in the tumor environment, hypoxia-induced factors spur the expression and release of growth factors that tip the equilibrium in favor of growth of new vessels to supply the tumor [7, 8]. Some of the growth factors implicated in tumor vascularization include vascular endothelial growth factor (VEGF), placental growth factor (PlGF), fibroblast growth factor (FGF) and hepatocyte growth factor (HGF) [9, 10]. These growth factors employ plasma membrane-bound receptor tyrosine kinases (RTKs) for signaling to intracellular effectors [11]. Signaling by these receptors requires pathway mediators such as protein kinases and monomeric G-proteins of the Ras family. These proteins regulate the signaling pathway by switching between the GDP-bound inactive state and the GTP-bound active forms [12]. These conformational inter-conversions are aided by two types of proteins; the guanine nucleotide exchange factors (GEFs) and the GTPase-activating proteins (GAPs) that speed up the exchange of GTP for GDP and GTP hydrolysis to GDP, respectively [13]. Mutations of members of this family of proteins, as observed in K-Ras, abrogates the intrinsic GTPase activity leading to constitutive signaling and resistance to cancer therapies that target the upstream growth factors and RTKs [14, 15]. In other cancers, some of the G proteins such as RhoC are overexpressed and hyperactive [16]. The G-proteins are involved in various facets of cellular activities such as cell proliferation, differentiation, vesicular trafficking, and cytoskeletal organization [17]. The Rho subfamily such as Rho, Rac and Cdc42 not only mediate Ras downstream signals but are also involved in F-actin organization resulting in the formation of lamellipodia, filopodia and stress fibers. Actin filament assembly and disassembly is critical for controlling cell shape and movement [18]. It appears, therefore, that disrupting the functions of the monomeric G-proteins would curtail growth-promoting signaling from RTKs as well as alter the cytoskeletal organization that would be involved in the migratory processes of cancer cells in metastasis as well as in neovascularization.

The clinical management of cancer with antiangiogenic therapies has not kept pace with our understanding of the angiogenesis in tumors. Antiangiogenic therapies have involved targeting VEGF signaling with monoclonal antibodies that prevent VEGF binding to its receptor [19], engineered soluble components of the VEGF receptor that bind to and prevent VEGF from the RTK enzyme domains [20]. Development of resistance invariably occurs upon treatment with antiangiogenic drugs [21]. Evasive mechanisms involve, amongst others, the increased expression of the growth factors [21, 22]. Therefore, new antiangiogenic drugs are needed to add to the present arsenal to be used either as alternative therapies and/or in combination with current drugs as well as fill the therapeutic vacuum caused by the development of resistance.

The pertinent roles played by the monomeric G-proteins in growth factor signaling in general and F-actin organization, cell shape and motility in particular, offer a valid alternative approach for the development of a new class of pharmacological entities. These can both suppress tumor growth through direct inhibition of growth signaling and angiogenesis. While drugging of aberrant G-protein functions has been a major challenge in cancer drug development for a long time, newer approaches that target the secondary modifications essential for the proteins to function has shown some promise in *in vitro* and *in vivo* experiments. K-Ras is typically farnesylated while the Rho subfamily members are geranylgeranylated [23, 24]. The last step in the polyisoprenylation pathway for these modifications involves enzyme-catalyzed polyisoprenyl-dependent methyl esterification and de-esterification reactions believed to add another level of control of the G-proteins' activities [25–27]. In our previous studies, we have found that inhibiting the esterase in various cancer cell lines induces apoptosis, inhibits cell migration, disrupts F-actin and alters cell shapes and sizes [28–30]. Based on the aforementioned studies, we developed the polyisoprenylated cysteinyl amide inhibitors (PCAIs) for the esterase which were far more effective against various biological processes that promote cancer [31]. Thus, the pertinent question then arose to find out if the PCAIs may be antiangiogenic given their effects on cell migration and F-actin. Here, we show that the PCAIs inhibit tube formation and angiogenesis in chick CAM and zebrafish embryos at submicromolar concentrations. These findings point towards the potential therapeutic application of the PCAIs in the management of cancer.

## RESULTS

### PCAIs inhibit HUVEC network capillary-like tube formation and *in vitro* angiogenesis

**Figure 1:**
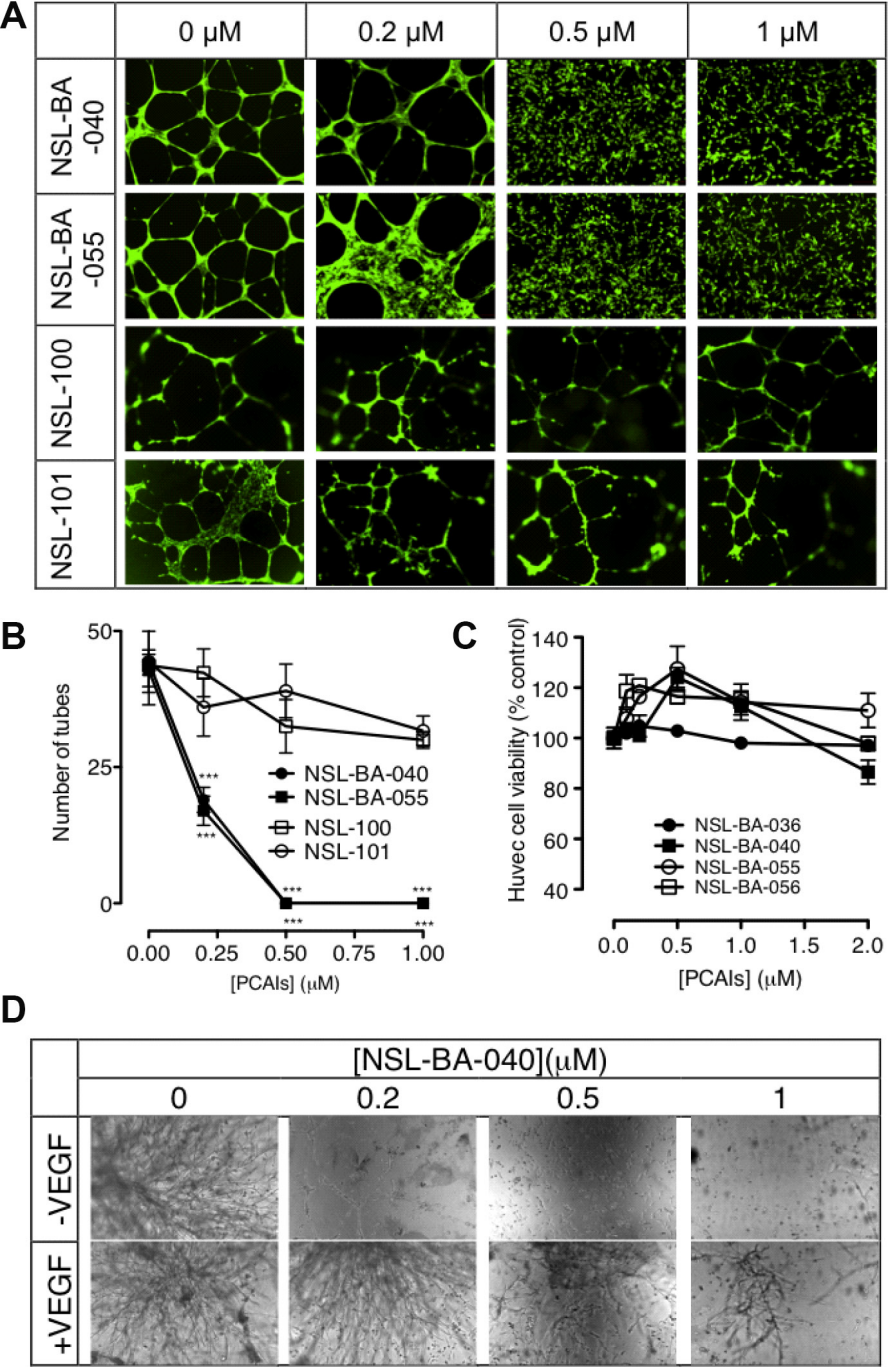
PCAIs inhibit HUVEC tube formation and *in vitro* angiogenesis but not HUVEC cell viability (**A**) HUVEC cells were plated on Matrigel in 24-well plates and treated with (0 – 1 μM) of NSL-BA-040, NSL-BA-055, NSL-100 and NSL-101 for 16 h. Cells were washed with DPBS containing calcium and magnesium and stained with 2 μg/mL Calcein AM before being imaged and analyzed as described in the methods. Representative annotated images showing areas of thick cells, tubes, loops, and branching points are shown. (**B**) The number of tubes formed in each treatment was quantified by counting the connected cells in ten randomly selected fields using NIH ImageJ software. (**C**) HUVEC cell viability was determined after 48 h of treatment with the PCAIs as described in the methods. Each data point represents the mean ± SEM, *N* = 4. (**D**) HUVEC cells with or without added VEGF in complete growth medium (LVES-supplement Medium 200) were seeded between two layers of LDEV-Free Reduced Growth Factor Basement Membrane Matrix Matrigel as described in the methods, and imaged after 48 hours. Nikon Eclipse Ti inverted fluorescent microscope equipped with Nikon monochrome digital camera at 10× magnification was used for all imaging and representative images were analyzed. For each assay, four independent experiments were done in triplicates. Significance (***p < 0.001) was determined by one-way ANOVA, followed by the Dunnett's post-test.

Tumor angiogenesis is a critical step in tumor progression and metastasis. The capillary-like tube formation is the result of a dynamic remodeling process of the vascular system [32]. Therefore, blocking tumor-induced angiogenesis and/or normalizing the tortuous tumor vasculature represents a strategic option for cancer treatment and prevention. When HUVECs were treated with or without the PCAIs for 16 to 18 hours, we observed a concentration-dependent inhibition of the capillary-like tube formation. The controls revealed that the endothelial cells grown on the Geltrex LDEV Matrigel organized into a branching network of capillary tube-like structures composed of multiple cells with intercellular spaces or lumens as shown on Figure 1A. The images clearly showed prominent areas covered with thick walls of cells, loops and branching points [33]. Treatment with NSL-BA-040 and NSL-BA-055 (0.2 μM) resulted in more thick walls of cells, less tubes, less loops, and less branching points. A slight disruption of bridges and branching points is seen following treatment with 0.2 μM NSL-BA-040. Treatment with 0.5 and 1 μM completely blocked the formation of tube-like structures. Specifically, no development of lumens in the cell-cell connections was noticed. The inhibitory effect on the HUVECs tube formation shown on Figure 1B reveals more than 2-fold reduction in the number of tubes following treatment with 0.2 μM PCAIs. Virtually all of the tube formation was abrogated at 0.5 μM. This was evident by the presence of confluent cell-covered areas in the absence of tube-like structures. As shown in Figure 1A and 1B, the non-farnesylated NSl-100 and NSL-101 did not prevent the formation of tubes even at 1 μM. Comparable numbers of tubes and branching points of identical quality and quantity could be seen in the NSL-100- and NSL-101 -treated as in the controls.

Since the PCAIs have been shown to cause apoptosis in cancer cells, albeit at higher concentrations [28], it was imperative to determine whether some or all of the anti-angiogenic effects could be due to cytotoxicity. When the HUVEC cells were treated with up to 2 μM of the PCAIs, no significant effects on cell viability relative to controls were observed (Figure 1C).

The *in vitro* angiogenesis or the *in vitro* sandwich tube formation assay showed a quantitative variation of capillary-like tube formation. It was noticed that in the presence or absence of VEGF, the NSL-BA-040 inhibited the sprouting of tube-like structures in a concentration-dependent manner. As shown in Figure 1D, the effect of this compound was even more pronounced in treatments without any VEGF. The relative number of capillary-like tubes formed decreased as the concentration of the PCAIs was increased. NSL-BA-040 almost totally inhibited the capillary-like tube formation in the absence and presence of VEGF-stimulated HUVEC at the sub-micromolar concentrations of 0.2 and 0.5 μM.

### PCAIs inhibit FGF- and VEGF-induced HUVEC migration and invasion

**Figure 2:**
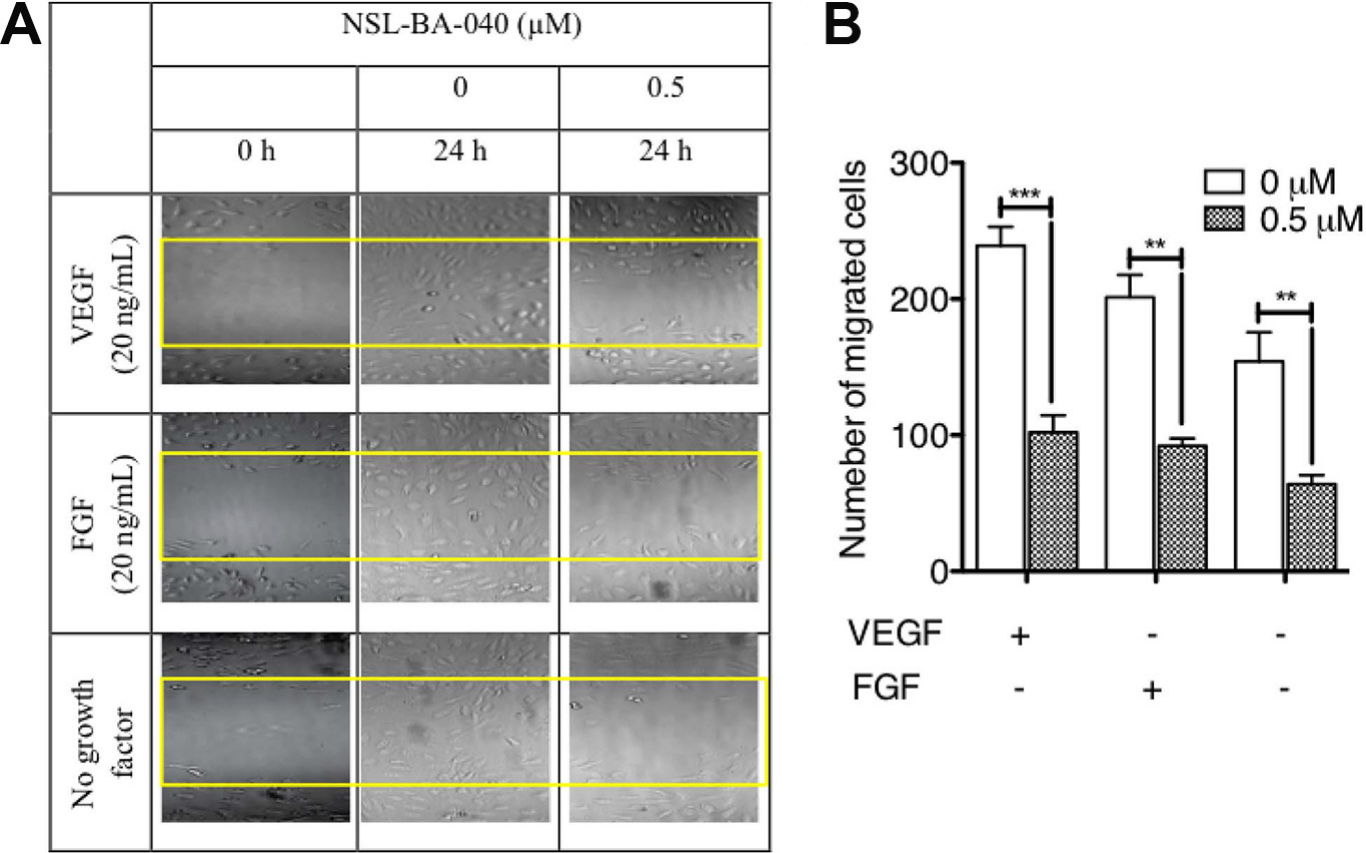
PCAIs inhibit HUVEC migration *in vitro* The effect of PCAIs on HUVECs migration was determined using the wound healing method as described in the methods. Wound closures were captured at 10× magnification using the Nikon Eclipse *Ti* inverted fluorescent microscope. (**A**) Representative images of three independent experiments conducted in quadruplets are shown. (**B**) The average number of migrated cells was quantified and analyzed using NIH ImageJ software. The plots are of the means ± SEM, *N* = 4. Significance (**p < 0.01 ***p < 0.001) was determined by one-way ANOVA followed by the Student's *t*-test.

**Figure 3:**
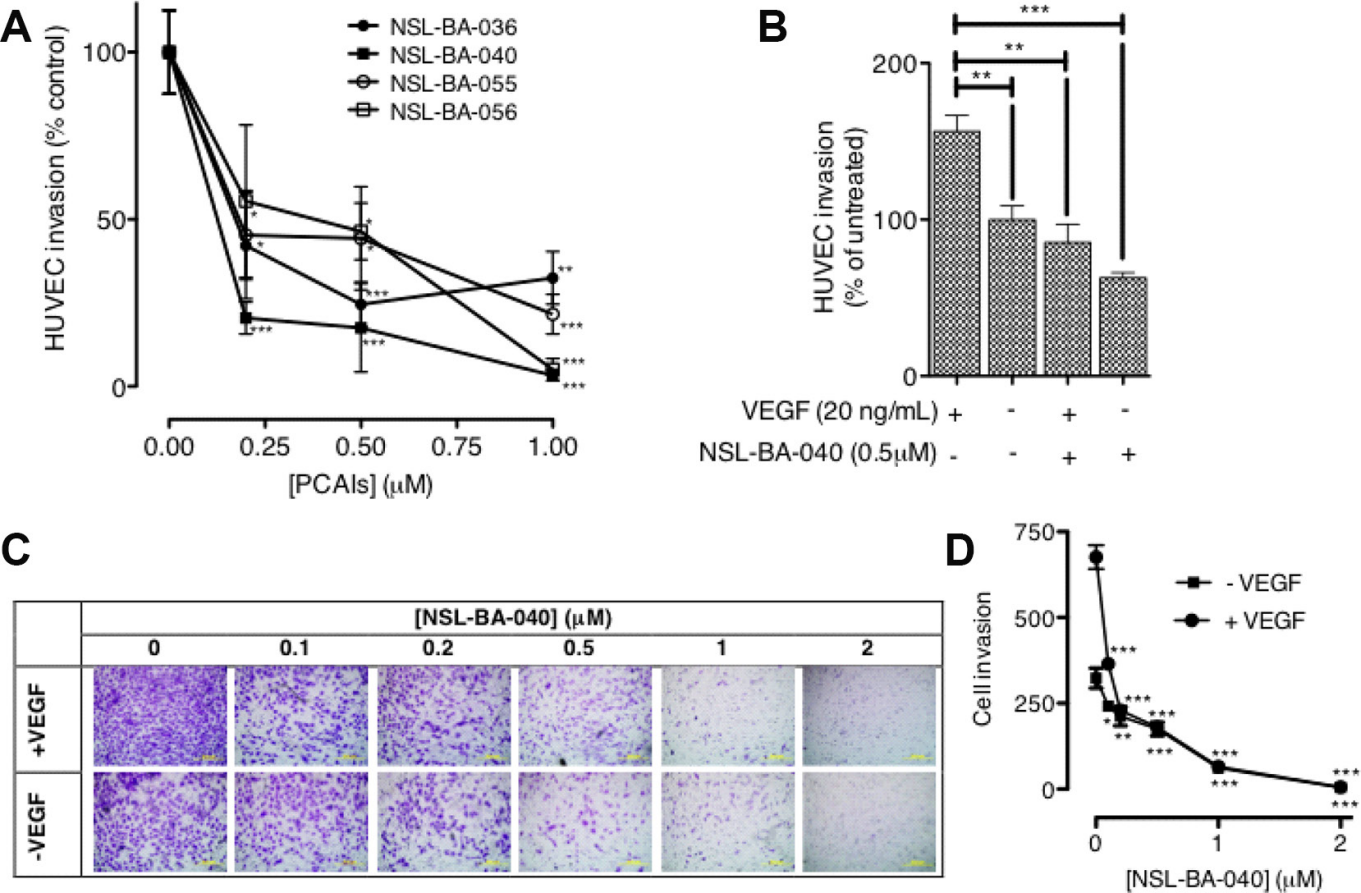
PCAIs significantly inhibit HUVEC migration and invasion (**A**) HUVEC cells were plated and treated with PCAIs as described in the methods. Invaded cells were stained with calcein AM and the fluorescence was read. The mean percentages ± SEM, *N* = 4 of invaded cells compared to the untreated control was determined and plotted. (**B**) VEGF HUVEC-stimulated cells were plated, treated with NSL-BA-040 and stained with calcein AM as described in the methods. The mean percentages ± SEM, *N* = 4 of invaded cells compared to the untreated control was determined and plotted. (**C**) Native and VEGF- stimulated HUVEC cells were plated, treated with NSL-BA-040, stained with crystal violet and imaged as detailed in the methods. Representative images of three independent experiments done in triplicates are shown. (**D**) The invasion plot for the relative number of invaded cells ± SEM, *N* = 4 was analyzed and plotted. Significance (*p < 0.05, **p < 0.01, and ***p < 0.001) was determined by one-way ANOVA followed by the Dunnett's post-test.

Since the PCAIs blocked capillary-tube formation and *in vitro* angiogenesis but did not show any significant effects on HUVEC cell viability, we next wanted to know whether the PCAIs could inhibit FGF- and VEGF-stimulated HUVEC cell migration and invasion. To achieve this, we determined the potential of PCAIs to inhibit native as well as FGF- and VEGF-stimulated HUVEC cell migration in the wound healing and Transwell assays. As shown in Figure 2A, NSL-BA-040 inhibited over 50% of HUVEC cell migration into the wound area in both FGF- and VEGF-stimulated but untreated HUVEC cells compared to the VEGF-stimulated HUVEC cells that showed a robust number of migrated cells in to the wound area of (Figure 2B). To further confirm observations in the wound-healing assay, we tested the effect of the compound on the Transwell monolayer permeability assay. As shown in Figure 3A, the PCAIs demonstrated consistent and significant increasing inhibition of the cell invasion. More interestingly, these PCAIs were far more potent at inhibiting HUVEC invasion than Sulforaphane, which inhibited only 11 and 31% of the invasion at 5 and 10 μM, respectively (Supplementary Figure S1). The NSL-BA-040 at 1.0 μM, inhibited HUVEC invasion by more than 10-fold compared to the control. Even in the presence of VEGF (Figure 3B), 0.5 μM of NSL-BA-040 significantly inhibited HUVEC cell migration and invasion through the Matrigel in the Transwell invasion assays. Figure 3C and 3D show that large numbers of VEGF-stimulated HUVEC cells migrated through the Matrigel to the lower side of the Corning Transwell compared to the non-stimulated controls. However, following treatment with NSL-BA-040, it was observed that the number of cells that migrated and invaded the lower chamber was significantly inhibited in a concentration-dependent manner compared to the untreated controls. Figure 3D reveals a 3-fold inhibition of VEGF-stimulated cell migration and invasion by 0.5 μM of NSL-BA-040.

### PCAIs disrupt neovasculization in the CAM angiogenesis model

**Figure 4:**
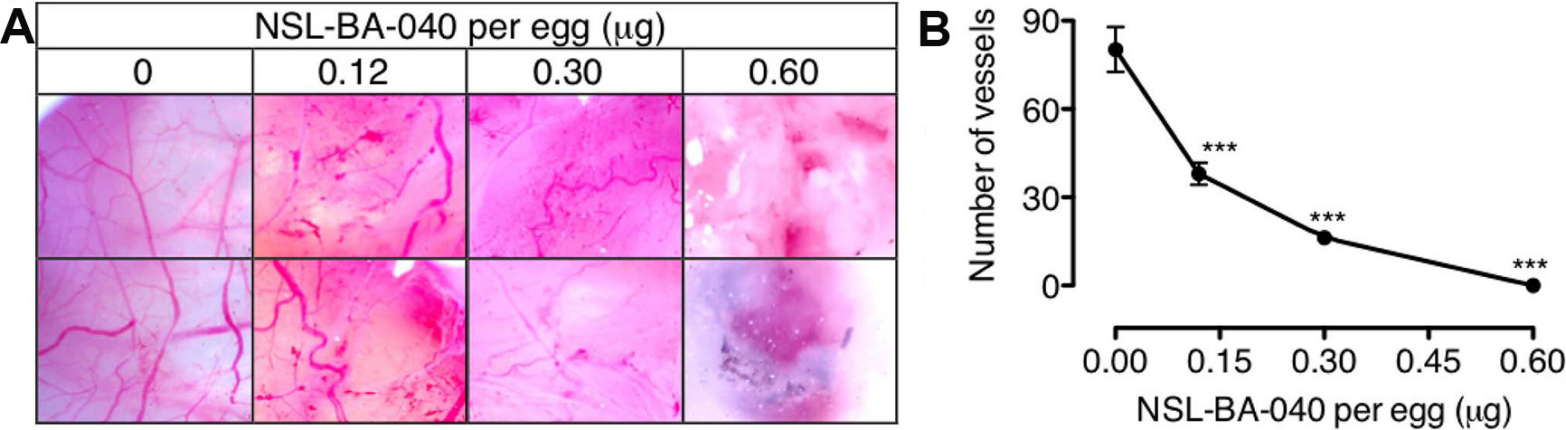
PCAIs disrupt neovasculization in the CAM angiogenesis assay Eggs were treated from the 8th day post-fertilization with NSL-BA-040 (0–0.60 μg) for 48 hours as described in the methods. (**A**) A section of the shell of each egg was removed and the degree of vascularization documented using Biology ProScope H system. (**B**) The number of vessels in 10 random areas per treatment was determined using NIH ImageJ software. The results are the means ± SEM, *N* = 4. The significance (***p < 0.001) was determined by one-way ANOVA, followed by the Dunnett's post-test.

The chick chorioallantoic membrane (CAM) is a very useful *in vivo* model for studying pro- and anti-angiogenesis agents [34]. Since the PCAIs inhibited HUVEC network capillary-like tube formation *in vitro*, we further investigated the potential of the PCAIs to limit cancer progression by determining its inhibitory potency in the CAM assay. As shown in Figure 4A, neovascularization was very conspicuous in the vehicle-treated fertilized egg with numerous vessel branches. The number of vessels significantly decreased in a concentration-dependent manner when the fertilized eggs were treated with NSL-BA-040 for two days, that is, on the eighth day post-fertilization. The number of vessels and branches decreased 2-fold compared to the control when 0.12 μg of NSL-BA-040 was added to the opened window of each egg (Figure 4B). There were virtually no vessels when the eggs were treated with 0.60 μg of the PCAIs.

### PCAIs inhibit angiogenesis in zebrafish

**Figure 5:**
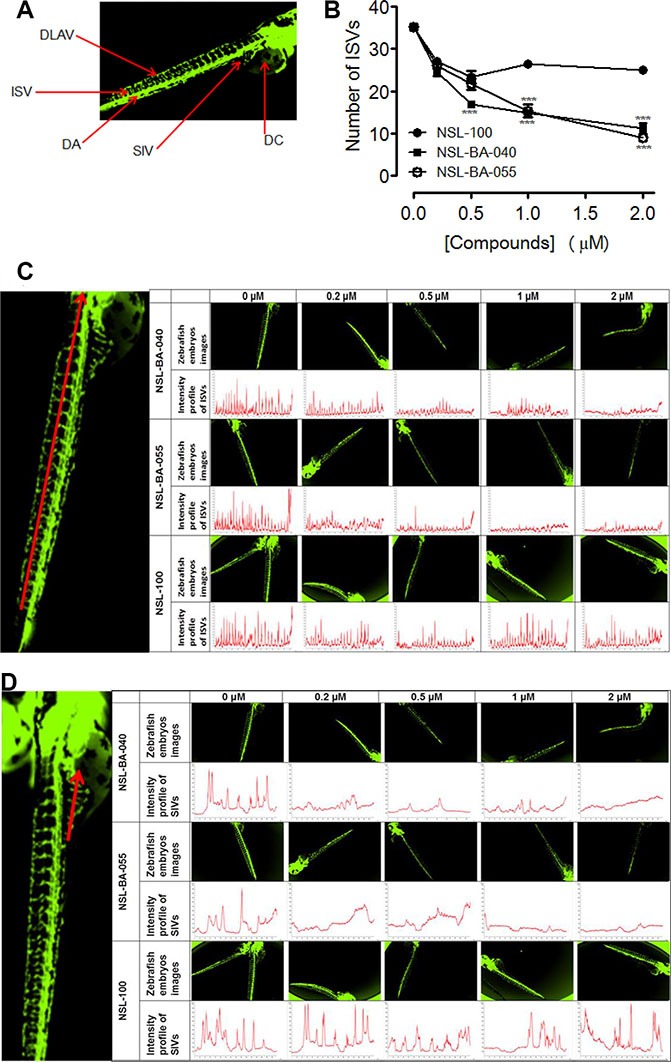
PCAIs disrupt the formation of zebrafish intersegmental vessels (ISVs) and subintestinal veins (SIVs) Transgenic (fli1: EGFP) Zebrafish embryos, 24 hpf were treated with NSL-BA-040, NSL-BA-055 and NSL-100 for 48 h and imaged as described in the methods. (**A**) Zebrafish anatomic regions highlighting angiogenesis targets: intersegmental vessels (ISV), dorsal longitudinal anastomotic vessel (DLAV), dorsal aorta (DA), subintestinal vein (SIV) and duct of cuvier (DC). (**B**) Zebrafish ISVs plots of the means ± SEM, *N* = 6–8. Significance (**p < 0.01 and ***p < 0.001). (**C**) and (**D**) Representative images and intensity profiles of zebrafish ISV and SIV, respectively depicting the effect of the compounds. The peak analysis for each compound was fixed based on the intensity of its untreated control.

Zebrafish has evolved into one of the most extensively used models of the vertebrate system for anti-angiogenesis drug screening. We investigated the effects of PCAIs and the non-farnesylated compound, NSL-100 on the angiogenic process in zebrafish embryos. Our data (Figure 5A and 5B) show that PCAIs inhibited angiogenesis in zebrafish embryo after 48 h treatment. The average number of ISVs at the end of treatment for the untreated controls was 35 ± 1. On the contrary, the number of ISVs in embryos exposed to 0.2 μM of each test compound were 27 ± 1, 25 ± 0 and 27 ± 1 for NSL-100, NSL-BA-040 and NSL-BA-055, respectively. The numbers reduced significantly to14 ± 0 and 15 ± 1 for NSL-BA-040 and NSL-BA-055, respectively but remained at 27 ± 0 for NSL-100 following exposure to 1 μM of each compound. The trend of the intensity profiles for the ISVs and SIVs of the untreated embryos and those treated with NSL-BA-040 and NSL-BA-055 showed concentration-dependent decreases in the intensities of ISV (Figure 5C) and SIV (Figure 5D). Similarly, the intensity profiles of ISVs and SIVs were not clearly demarcated in NSL-100-treated samples as seen in Figure 5C and 5D. The number of distinct fully integrated peaks depicting ISVs decreased from the untreated control in a concentration-dependent manner following treatment with NSL-BA-040 and NSL-BA-055. This was not the case for compound NSL-100 that showed almost identical profiles with the untreated controls. We also observed a progressive drop of peak intensities of ISVs to about 700 for embryos treated with 2 μM of NSL-BA-040 or NSL-BA-055 compared to the untreated embryos. The peak intensities for untreated embryos and those treated with NSL-100 showed almost similar trends (Figure 5C). The intensity profiles for the SIVs followed a similar trend to those observed for the ISVs.

## DISCUSSION

The *de novo* formation of blood vessels is an active normal physiological process in embryogenesis but also occurs in adults during wound healing, tissue regeneration, and the female reproductive cycle [3, 35]. It is a tightly regulated process by pro- and anti-angiogenic factors that are normally present in the body. In response to abnormal changes in their microenvironment, the pro-angiogenic factors predominate in many disease conditions including cancer, osteomyelitis, arthritis, atherosclerosis and ovarian cysts [36]. Angiogenesis is essential for tumors that cannot grow beyond 200–300 μm in diameter unless new blood vessels are formed to supply them with nutrients and oxygen [37]. Angiogenesis is therefore very critical for cancer growth, survival and metastasis [37, 38]. The mortality rate of pancreatic cancer approaches 100% due to rapid metastatic transformation that is aided by angiogenesis [39]. Therefore, designing and developing compounds that target and disrupt pathogenic angiogenesis is thus a subject of great interest especially for cancer therapy [38–41].

We previously detected profound effects of PMPMEase inhibition using L-28 on F-actin organization [30]. Since the polyisoprenylated proteins, Rho, Cdc42 and Rac are at the center of lamellipodia, filopodia and stress fiber formation necessary for cells to migrate, we wanted to know whether the PCAIs may, by disrupting F-actin organization, inhibit the arrangement of cells leading to tube formation during angiogenesis. It was thus interesting to observe the profound effects of the PCAIs against HUVEC tube formation as well as vessel formation in CAM and zebrafish embryos. Although the exact mechanisms of these observations is not clear since the direct molecular targets of the PCAIs are yet to be identified, the fact that the non-polyisoprenylated analog NSL-100 did not show similar effects suggests interference with polyisoprenylation-dependent interactions. Polyisoprenylated cysteine-specific binding sites have long been detected in some proteins that modulate the effects of polyisoprenylated proteins. Rho dissociation inhibitors (RhoGDI), that inhibit the activities of Rho family proteins, are known to harbor a polyisoprenyl cysteine-binding site [42, 43]. The insertion of the polyisoprenyl moiety of Cdc42 into the hydrophobic pocket of RhoGDI was determined to be the rate-limiting step for Cdc42 extraction from membranes [43]. Studies with geranylated, farnesylated and geranylgeranylated thiosalicylic acid revealed the geranylated and geranylgeranylated analogs to be the least and most potent at inhibiting the interactions of RhoGDI with various Rho family proteins [44]. Although it is yet to be determined whether the PCAIs interact with RhoGDIs and whether any such interactions will lead to changes in F-actin organization, the effects of the related compounds and the polyisoprenyl binding pockets in RhoGDIs strongly suggest this possibility. Cell motility necessary for cell invasion has been inextricably linked to the F-actin-based projections driven by Rho family proteins [18].

Rho family proteins also play intricate roles in downstream signaling by receptor tyrosine kinases (RTKs). For example, the vascular endothelial growth factor (VEGF) receptor signaling activates Rho, Rac and Cdc42 [45, 46]. That the PCAIs were able to drastically suppress both basal and VEGF-stimulated HUVEC cell invasion of Matrigel is an indication of possible disruption of signaling events downstream of the VEGF receptor. This is consistent with the observed inhibition of basal as well as the FGF- and VEGF-stimulated cell migration and invasion. Various polyisoprenylated proteins are involved in downstream signaling by these receptors. In addition to the aforementioned G proteins, other G proteins of the monomeric types such as Ras as well as heterotrimeric G proteins, of which the γ–subunits are polyisoprenylated, are involved [46]. The fact that these anti-cell migratory, anti-invasive and antiangiogenic effects occurred at concentrations that were significantly lower than the EC_50_ values against cell viability [31] is an indication that the observed effects may not be due to cytotoxicity-induced immotility of the cells but most likely a perturbation of one or more polyisoprenylated protein cytoskeletal function, the identities of which need to be investigated. Furthermore, these effects were observed at incubation periods significantly shorter than typically required for apoptosis to occur in various cancer cell lines.

The ability to successfully block angiogenesis is likely to accrue numerous benefits for therapeutically effective cancer therapies. In the absence of blood vessels to serve growing tumors, nutrients and oxygen supply to the tumors will be significantly diminished thereby limiting tumor growth. Furthermore, since metastasis also relies on the blood vessels for the budding cancer cells to escape into the general circulation to eventually implant into distal sites and grow into secondary tumors [47], inhibiting angiogenesis is likely to negatively impact the spread of the cancer and constitute a powerful avenue for therapeutic mitigation of malignancy. Also, angiogenesis, while being a widespread and necessary process in embryonic development, it occurs only in a few adult physiological events such as in wound healing [4], uterine thickening in the menstrual cycle [5] and vascularization in the eye resulting in loss of vision [48]. It appears therefore that the PCAIs may serve a very selective cancer therapeutic benefit by virtue of inhibiting a physiological process that is not essential for day-to-day survival. That the PCAIs can exert this effect at significantly sub-cytotoxic concentrations bodes well for their potential therapeutic safety. This is further substantiated by their ability to inhibit angiogenesis in zebrafish embryos at non-lethal concentrations.

## MATERIALS AND METHODS

### Materials

**Figure 6:**
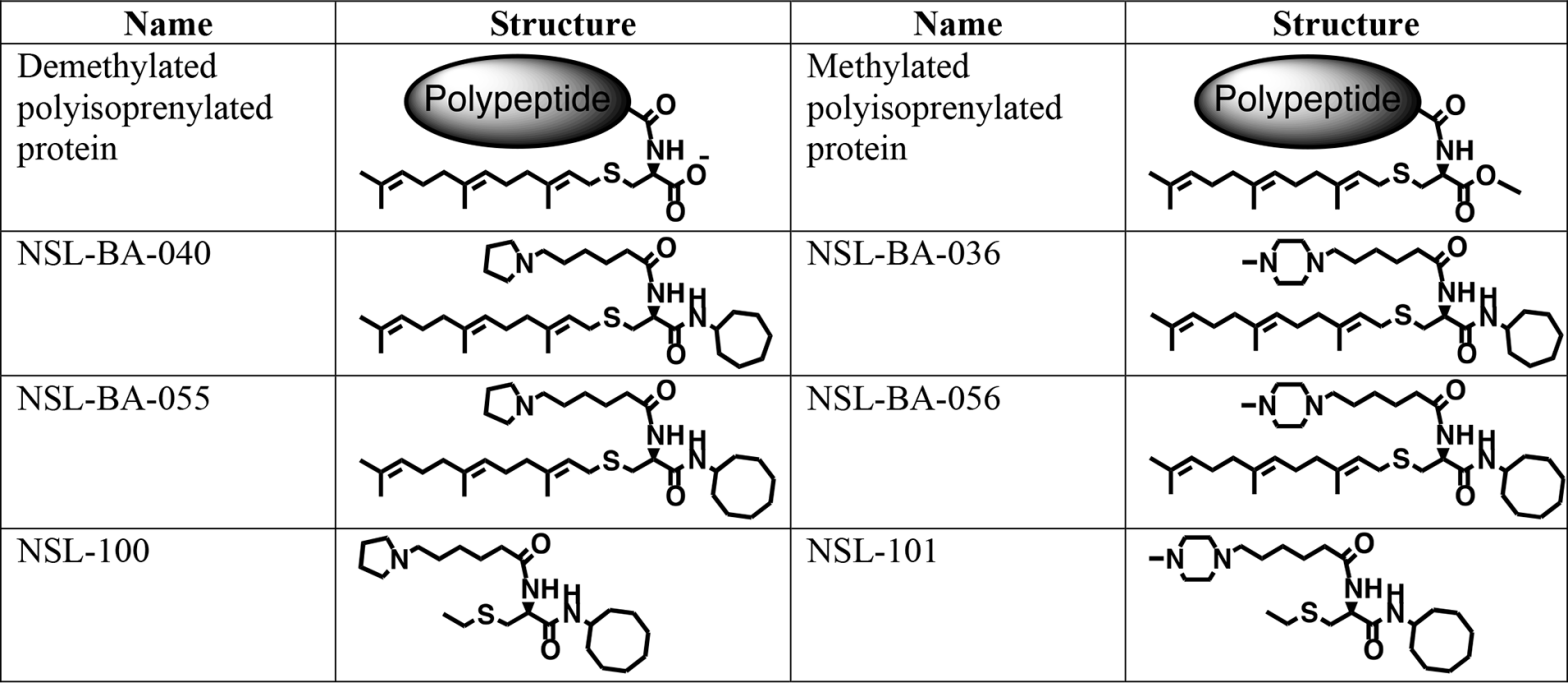
Shown are the chemical structures of the PCAIs and their non-farnesylated analogs in relation to the secondary modifications on polyisoprenylated proteins.

The PCAIs (Figure 6) were synthesized as previously described [31]. The *in vitro* angiogenesis starter kit consisting of human umbilical vein endothelial cells (HUVEC), large vessel endothelial supplement (LVES, 50×), Medium 200 and Geltrex lactate dehydrogenase-elevating virus (LDEV)-free reduced growth factor basement membrane matrix were obtained from ThermoFisher Scientific (Waltham, MA) and used for the studies within two months of purchase. Human vascular endothelial growth factor (VEGF) and human fibroblast growth factor (FGF) were purchased from Life Technologies (Grand Island, NY). Penicillin (100 U/mL) and streptomycin (100 μg/mL) were obtained from Atlanta Biological (Atlanta, GA). Cultrex *in vitro* angiogenesis assay endothelial cell invasion kit was supplied by Trevigen (Gaithersburg, MD). Fertilized chicken eggs were purchased from Johnnie's Garden (Quincy, FL). Zebrafish embryos were purchased from Zebrafish International Research Center (ZIRC), University of Oregon (Eugene, OR).

### Effect of PCAIs on capillary-like tube formation and *in vitro* angiogenesis

The effect of PCAIs on capillary-like tube formation and on *in vitro* angiogenesis was determined using the *in vitro* angiogenesis starter kit. HUVEC cells were cultured in a 5% CO_2_/95% humidified airflow incubator maintained at 37°C using LVES-supplement Medium 200 prepared by adding 11 mL LVES and 5 mL penicillin/streptomycin. HUVEC cells were cultured by seeding 2500 cells per cm^2^ in T-75 flasks containing LVES-supplement Medium 200. The growth medium was replaced every two days until the sixth day when the cells were about 80 to 90% confluency. Geltrex LDEV-free reduced growth factor basement membrane matrix Matrigel (0.1 mL) was transferred to each well of the 24-well plate and incubated for 30 min at 37°C in a 5% CO_2_/95% humidified airflow incubator. To test the effect of PCAIs on tube formation, 50000 cells in LVES-supplement Medium 200 were plated on the polymerized Geltrex LDEV Matrigel surface layer. The cells were immediately treated with the respective PCAIs and incubated for 16 h under 5% CO_2_/95% humidified airflow at 37°C. The cells were washed with Dulbecco's Phosphate-Buffered Saline (DPBS) containing 0.90 mM calcium chloride and 0.49 mM magnesium chloride followed by treatment with 2 μg/mL Calcein AM. Then, incubated for an additional 30 min before images of the cells were captured using a Nikon Eclipse Ti microscope at 10× magnification. Independent experiments conducted in triplicates were repeated four times. The results were quantified by randomly capturing images in 10 different spots per well. The number of tubes formed per well was quantified using NIH ImageJ software by randomly choosing 10 different images from each well and counting the numbers of tubes formed.

To test for the effect of PCAIs on *in vitro* sandwich tube formation assay, HUVEC cells (50000 /well) were seeded in each well of a 24-well plate on the surface polymerized Geltrex LDEV Matrigel with LVES-supplement Medium 200 supplemented with either 20 ng/mL VEGF or FGF. A second layer of the Geltrex LDEV Matrigel was added on to the seeded cells. Each well was treated with the PCAIs (0 – 1 μM) and returned into the 5% CO_2_/95% humidified airflow incubator maintained at 37°C. After 48 hours of incubation, the images were captured at 10× magnification using Nikon Eclipse *Ti* inverted fluorescent microscope equipped with Nikon monochrome digital camera.

### Effect of PCAIs on HUVEC viability

HUVEC cells were cultured to about 90% confluency in LVES-supplement Medium 200, harvested and plated on 96-well plates at a density of 20,000 cells per well in 100 μL LVES-supplement Medium 200. After 24 hours, the cells were treated twice with concentrations of PCAIs ranging from 0 – 2 μM. After 24 and 48 hours, the viability of the cells was determined using the resazurin reduction assay as previously described [28].

### Effect of PCAIs on HUVEC migration and invasion

The wound-healing assay was conducted to measure the effect of PCAIs on unidirectional cell migration. The HUVEC cells cultured in LVES-supplement Medium 200 to about 90% confluent were harvested and plated at a density of 5 10^5^/well into a 24-well plate with the IBIDI inserts (Mt. Prospect, IL) followed by incubation at 37°C in a humidified 5% CO_2_ incubator. After 24 hours of incubation, the cells formed a fully confluent monolayer and the inserts were carefully removed using forceps. The cells were washed with DPBS and starved by incubating in 2% LVES-supplement Medium 200 for 6 hours. The 2% LVES medium was replaced with LVES-supplement Medium 200 alone or stimulated with 20 ng/mL of either VEGF or FGF and treated with or without NSL-BA-040 (0.5 μM). Images of wound closure were captured in phase contrast at 10× magnification using the Nikon Eclipse *Ti* inverted fluorescent microscope at 0, 6, 12 and 24 hours. The average number of cells that migrated into the wound area after 24 hours was counted and plotted using NIS-Element Nikon software.

The effect of PCAIs on HUVEC migration and invasion was further investigated using the Transwell assay. To achieve this, 50 μL of 0.1X basement extracellular membrane (BME) Matrigel was used in each well to coat the top chamber of the 96-well invasion device followed by incubation for 4 hours at 37°C in a humidified 5% CO_2_ incubator. The coating solution was then carefully aspirated from the top chamber. HUVEC, cultured to about 80% confluency were harvested, counted and diluted to 4 × 10^5^cells/mL in Medium 200 without LVES. Cells (20,000/well) suspended in 50 μL were plated on the surface of the BME Matrigel-coated top chamber. The cells were treated with concentrations of PCAIs ranging from 0 – 1 μM. Sulforaphane [1-isothiocyanato-(4R)-methylsulfinyl)-butane] (5 and 10 μM), provided in the kit was used as a positive control. LVES-supplement Medium 200 (150 μL/well) was added to the bottom chamber and the device was incubated for 24 and 48 hours at 37°C in a humidified CO_2_incubator. After the incubation, the top and the bottom chambers were washed with 100 and 200 μL of the provided 1 × wash buffer, respectively. Cell dissociation solution mixed with Calcein AM (150 μL) at a ratio of 1:1000 was added to each bottom chamber well and incubated for 1 hour. To ensure optimal dissociation, the device was gently tapped 10 times on the side midway during incubation period. The cell invasion device was disassembled and the relative fluorescence of the bottom chamber was determined at 485 nm excitation and 520 nm emission using Tecan fluorescence microplate reader (Morrisville, NC). The effect of PCAIs on cell invasion was calculated as a percentage of the treated versus the untreated controls.

To determine the effect of the PCAIs on the chemotactic effects of VEGF, 50,000 HUVECs were plated into each insert of the Corning Transwell chamber containing 0.5 mL medium lacking LVES followed by treatment with concentrations of NSL-BA-040 ranging from 0 – 2 μM. The bottom wells were filled with 0.75 mL 10% LVES-supplement Medium 200 with or without 20 ng/mL VEGF as chemo-attractant. The assembly was incubated for 24 hours at 37^°^C in a humidified 5% CO_2_ incubator. Non-penetrating cells that did not migrate were rinsed off with DPBS and removed from the surface of the filter with cotton swabs. Cells that migrated through the Matrigel into the underside of the membrane were fixed with 4% formaldehyde for 30 minutes and stained with 0.1% crystal violet solution for 1 hour. In order to quantify for cell invasion, a total of 20 images per well were randomly taken using an inverted fluorescent Olympus microscope equipped with a DP70 Camera set at 20× magnification. The number of migrated cells was counted using NIS-Elements Nikon software and plotted using GraphPad Prism software.

### Effect of PCAIs on chick chorioallantoic membrane (CAM) assay

The CAM assay was used as an *in vivo* model to test for the potential of the PCAIs to abrogate angiogenesis. Fertilized white and brown chicken eggs in groups of 24–30 were incubated in a humidified HOVA-BATO Incubator NO 1582 (Savannah, GA) at 37°C. On the eighth day of incubation, eggs were randomly placed in groups of six. A sterilized dissecting needle was used to open a very tiny hole through the outer shell of a sterilized area around the air space near the end of each egg. Using a sterilized insulin injection needle, NSL-BA-040 (0 – 0.6 μg) dissolved in 50 μL DPBS was injected into the air space. The Treatment procedure was repeated after 24 hours. The windows were resealed after each treatment with adhesive tape and the eggs returned to the incubator. After 48 hours following treatment, photographs of the exposed area were taken using the Biology ProScope H system (Beaverton, OR). The relative number of arteriole branches in each image was counted using NIH ImageJ software (http://rsb.info.nih.gov/ij/) [28, 49]. The experiment was repeated three times.

### Effects of PCAIs on angiogenesis in zebrafish embryos

Use of the zebrafish embryo model to study the effects of PCAIs on angiogenesis was approved by the Florida A&M University Institutional Animal Care and Use Committee. Transgenic Zebrafish embryos (fli1: EGFP) purchased from ZIRC were incubated at temperatures between 25−30°C in 200 μL of 0.5× E2 Medium (7.5 mM NaCl, 0.25 mM KCl, 0.5 mM MgSO_4_, 75 μM KH_2_PO_4_, 25 μM Na_2_HPO_4_, 0.5 mM CaCl_2_, 0.35 mM NaHCO_3_, 0.5 mg/L methylene Blue) [49] until the time of treatment. One, or in some cases, 2 embryos were dispensed per well in 96-well assay plates 24–30 hours post fertilization (hpf). The embryos were dispensed in 190 μL of the 0.5× E2 Medium per well. Compounds (10 μL) prepared in Dulbecco's PBS were used for the treatment with the indicated concentrations, resulting in total final volumes of medium per well of 200 μL. After 24 to 48 hours of treatment, embryos were held in 30% Danieu's buffers containing tricaine methanesulfonate (final concentration 0.02%) [50] and imaged using a Nikon Eclipse *Ti* inverted fluorescent microscope equipped with Nikon monochrome digital camera. The images were analyzed for the effects of the compounds on blood vessels formation. The vessels evaluated for the effects of the compounds included the dorsal longitudinal anastomotic vessel (DLAV), intersegmental vessels (ISV), dorsal aorta (DA) and subintestinal vein (SIV). The images were analyzed and quantified using NIS-Elements software. The total number of fully developed ISVs present in the trunk of each embryo of the untreated and treated embryos was counted. Prism GraphPad 5 was used to construct graphs and perform statistical analysis. The fluorescence intensity profile of the ISV (across midway of interconnected luminal pathways) and SIV (measurement taken at midway across the basket-like structure stretching into the posterior DC) of treated and untreated zebrafish embryos were also analyzed using the NIS-Elements software.

### Data analysis and statistics

The statistical analysis and graphical analysis of the data was performed using Prism (GraphPad 5 software) for EC_50_ determination (Non-linear regression, sigmoidal dose-response with variable slope). Data are presented as means ± standard error of mean (SEM). Significance (*p < 0.05, **p < 0.01, and ***p < 0.001) was determined by one-way ANOVA followed by the Dunnett's post-test to compare each data point with vehicle control.

## SUPPLEMENTARY MATERIALS FIGURES


