# Supplementary material for: The antiangiogenic effects of polyisoprenylated cysteinyl amide inhibitors in HUVEC, chick embryo and zebrafish is dependent on the polyisoprenyl moiety
Source: Oncotarget. 2016 Sep 8;7(42):68194–205. doi: 10.18632/oncotarget.11908 (PMC5356549; doi:10.18632/oncotarget.11908)
Supplement: Supplementary file 1 [file oncotarget-07-68194-s001.pdf]

The antiangiogenic effects of polyisoprenylated cysteinyl amide inhibitors in HUVEC, chick embryo and zebrafish is dependent on the polyisoprenyl moiety

Supplementary Materials

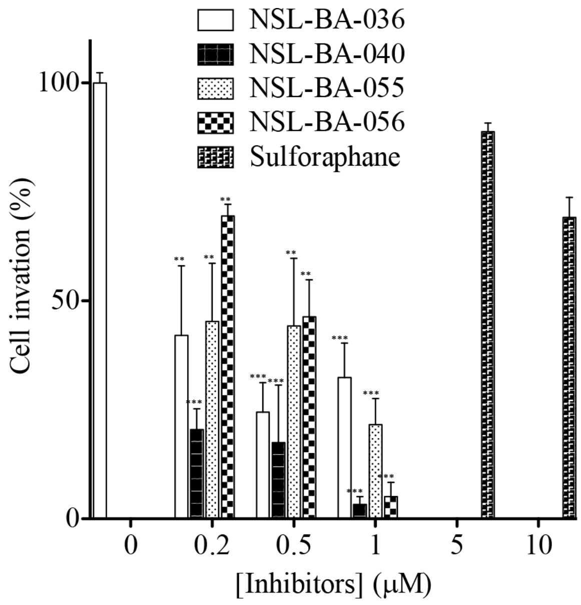

Supplementary Figure S1: PCAIs are significantly more potent inhibitors of HUVEC migration and invasion than Sulforaphane. HUVECs were plated and treated with PCAIs or sulforaphane as described in the methods. Invaded cells were dissociated and stained with dissociation solution mixed with Calcein AM (150 μL) at a ratio of 1:1000 added to the bottom chamber wells and incubated for 1 hour. To ensure optimal dissociation, the device was gently tapped 10 times on the side midway during the incubation period. The cell invasion device was disassembled and the relative florescence unit (RFU) of the bottom chamber was determined at 485 nm excitation and 520 nm emission wavelengths using Tecan fluorescence microplate reader (Morrisville, NC). The effect of PCAIs on cell invasion was calculated as a percentage of the treated versus the untreated controls fluorescence readings after staining with calcein AM. The mean percentages ± SEM, $N = 4$ of invaded cells compared to the untreated control was determined and plotted. Note that the data for the PCAIs are the same as in Figure 4A. These were determined in the same experiment as for sulforaphane. Significance (*$p < 0.05$, **$p < 0.01$, and ***$p < 0.001$) was determined by one-way ANOVA followed by the Dunnett's post-test.
